# Global haplotype partitioning for maximal associated SNP pairs

**DOI:** 10.1186/1471-2105-10-269

**Published:** 2009-08-27

**Authors:** Ali Katanforoush, Mehdi Sadeghi, Hamid Pezeshk, Elahe Elahi

**Affiliations:** 1 Institute of Biochemistry and Biophysics, University of Tehran, Tehran, Iran; 2 National Institute of Genetics Engineering and Biotechnology, Tehran, Iran; 3 School of Computer Science, Institute for Studies in Theoretical Physics and Mathematics, Tehran, Iran; 4 School of Mathematics, Statistics and Computer Science, and Center of Excellence in Biomathematics, College of Science, University of Tehran, Tehran, Iran; 5 Department of Biology, College of Science, University of Tehran, Tehran, Iran

## Abstract

**Background:**

Global partitioning based on pairwise associations of SNPs has not previously been used to define haplotype blocks within genomes. Here, we define an association index based on LD between SNP pairs. We use the Fisher's exact test to assess the statistical significance of the LD estimator. By this test, each SNP pair is characterized as associated, independent, or not-statistically-significant. We set limits on the maximum acceptable proportion of independent pairs within all blocks and search for the partitioning with maximal proportion of associated SNP pairs. Essentially, this model is reduced to a constrained optimization problem, the solution of which is obtained by iterating a dynamic programming algorithm.

**Results:**

In comparison with other methods, our algorithm reports blocks of larger average size. Nevertheless, the haplotype diversity within the blocks is captured by a small number of tagSNPs. Resampling HapMap haplotypes under a block-based model of recombination showed that our algorithm is robust in reproducing the same partitioning for recombinant samples. Our algorithm performed better than previously reported models in a case-control association study aimed at mapping a single locus trait, based on simulation results that were evaluated by a block-based statistical test. Compared to methods of haplotype block partitioning, we performed best on detection of recombination hotspots.

**Conclusion:**

Our proposed method divides chromosomes into the regions within which allelic associations of SNP pairs are maximized. This approach presents a native design for dimension reduction in genome-wide association studies. Our results show that the pairwise allelic association of SNPs can describe various features of genomic variation, in particular recombination hotspots.

## Background

Analysis of Single Nucleotide Polymorphisms (SNPs) in the DNA of unrelated individuals revealed a block-like structure of haplotype variation along the human genome. Using the first available genome-wide data of SNPs on chromosome 21, Patil et al. [1] showed that in particular regions on the chromosome, the observed diversity of SNP haplotypes is less than the expected. Almost at the same time, a similar structure in haplotypes within a region of 103 SNPs on chromosome region 5q31 was reported by Daly et al. [2]. In the latter study, a block structure of haplotypes was revealed using a Hidden Markov Model for estimating recombination rates. This approach, unlike models based on haplotype diversity, incorporated a quantity measuring Linkage Disequilibrium (LD) between pairs of SNPs.

It is well known that effects such as population bottlenecks, geographic isolation, and natural selection can increase the extent of linkage disequilibrium in genomes [3]. However, in established large populations under random mating, haplotype blocks reflect mutation events in the ancestors of that population and recombination events along the chromosomes. Increased frequency of recombination events are likely to create genomic regions with haplotype blocks of small size. Based on HapMap data, Myers et al. [4] identified short genomic regions within the human genome in which the recombination rates are orders of magnitude higher than background levels. Such regions are called "recombination hotspots". Identification of hotspots of recombination and estimation of rates of crossover therein are important issues [5-7].

However, existing approaches that address these issues are generally complicated and computationally intensive. Haplotype blocks can rapidly provide rough estimations about hotspots of recombination. Knowledge of haplotype blocks has other applications as well. The block structure of chromosomes can be used in statistical approaches aimed at achieving better understanding of genetic features that differentiate ethnic groups [8-10]. The phasing problem in genotype analysis is typically solved by computational methods; one can reduce the computational cost by breaking the input genotype data into smaller units along boundaries of haplotype blocks. As a consequence, block partitioning is performed prior to phasing and other analysis in genomic sequence-based endeavors, including those incorporated in Haploview [11]. Similarly, Zhao et al. [12] proposed the use of block partition inside the PL-EM algorithm for the haplotype inference problem [13], as a substitute for the common PL technique.

One of the most important applications of SNP haplotype data is in regard to identification of disease causing genes. State-of-the-art sequencing technologies that provide large volume SNP data along with efficient statistical analyses; have made the use of haplotype data for gene identification a realistic goal. These statistical analyses usually incorporate time demanding reiterative tasks on large data sets. Clearly, the reduction of data volume by making use of haplotype blocks allows for more rapid and efficient analyses. B. Browning and S. Browning [14] presented a method for disease association studies based on haplotype blocks that relies on pairwise association between SNPs. Other approaches in disease association studies require samples of limited haplotype diversity [15-17].

In case-control association studies designed to identify disease causing genes, one of two strongly associated SNPs can be used as a "proxy" for testing association of the trait with the other. Grouping such associated "proxy" SNPs together compresses information to be used for case-control studies. Many strategies have been proposed to conduct case-control studies in an economic manner [18-20]. The key idea shared by all these approaches is that essentially all important information derived by analysis of association tests between each individual SNPs and phenotype of interest can be derived by analysis of a subset of SNPs, called tagSNPs. The most widely used method for tagSNP selection has been introduced by Carlson et al. [21]. Applying a threshold on *r*^2^, in each iteration of Carlson's method, the SNP included in the largest number of associated pairs is selected as a tagSNP.

Another common approach is in identification of haplotype-tagging SNPs (htSNPs). Here, the goal is to find the smallest subset of the SNPs (htSNPs) such that any single nucleotide difference between any two distinct haplotypes can be captured by an htSNP. Ding et al. [22] have shown that htSNP selection is notably effected by the manner in which haplotype blocks are defined. Zhang et al. [23] have incorporated several criteria for "candidate block" identification into their dynamic programming algorithm for finding the minimum number of htSNPs in an entire chromosome. While there are different criteria to limit regions of the genome as "potential blocks", most of them can be classified as measures of haplotype frequency or measures of SNP pair association (see Table 1). These two measures may seem equivalent at first sight, but in fact they quantify different features of the data. For example, "common haplotypes" feature haplotype frequencies, whereas |*D'*| quantifies allelic association of SNP pairs. In theory, and even in practice, it is possible that values of these two measures derived from the same data will define different haplotype blocks [24]. However, the extent to which haplotype diversity is captured when blocks are defined on the basis of association of SNP pairs remains to be established.

**Table 1:** Summary of haplotype block partitioning methods compared in this study.

abbr.	Method	Partitioning structure	Block definition	Block constraint	Software	Ref.
**HOT**	Hotspot	Local*	recombination hotspots	none	"precomputed results available by HapMap"	[4]
**MB**	Minimum block number	Global	minimum number of blocks	haplotype diversity	HapBlock v.3	[23]
**HB**	HapBlock	Global	minimizing total number of tagSNPs	haplotype diversity	HapBlock v.3	[23]
**MDL**	MDBlock	Global	minimum description length	haplotype diversity	MDBlock v.1	[43]
**GAM**	Four gamete test	Local	evidence of recombination	fourth gamete	Haploview v.4	[44]
**GAB**	Gabriel's method	Local	evidence of recombination	strongly associated SNPs	Haploview v.4	[26]
**GPG**	GPMAP** based on Gabriel's index	Global	maximizing associated SNPs	independent SNPs	Haploview+GPMAP	present paper
**GPF**	GPMAP based on Fisher's exact Test	Global	maximizing associated SNPs	independent SNPs	Haploview+GPMAP	present paper

* The approach of the original method is recognized as a local partitioning. ** Global Partitioning for Maximal Association Pairs

In this work, we discuss on a haplotype block partitioning that is based on pairwise association of SNPs. There are several statistics for determining allelic correlation between SNPs. Two well known statistics are *D *and *r*^2 ^which, respectively, represent the sample covariance and the sample correlation coefficient of two bi-allelic markers. Theoretical distributions of these statistics have been well studied. For instance, it is known that *nr*^2 ^is asymptotically chi-squared [25], where *n *is the sample size. These two statistics depend on marginal frequencies of two SNPs. *D'*, a third measure of linkage disequilibrium introduced by Lewontin [25], is preferred by some researchers. *D' *is a normalized statistic with respect to marginal frequencies and varies linearly between 0 and 1 as a function of the forth gamete frequency.

In fact, although the point estimate of *D' *is independent of sample size, its distribution under the hypothesis of independence is a function of sample size. To measure the significance of *D'*, Gabriel et al. [26] suggested the use of an interval estimate. Here, we suggest that *p*-values as derived from the Fischer's exact test be used to assess the significance of *D'*. With respect to disease association studies, wherein this test is used to assess phenotype-SNP association, it seems reasonable to use the same test for SNP-SNP association.

Haplotype blocks can alternatively produce "local partitioning" and "global partitioning". In local partitioning, haplotype blocks are defined independently from the configuration of other haplotype blocks through the genome. Usually non-contiguous blocks are produced that look like a series of "islands" within the genome. In contrast, in global partitioning, the aim is to split the genome into haplotype blocks; the entire genome is "tiled" meaning all regions of the genome are within a block. Here, no single definition is applicable to all the blocks. To the best of our knowledge, little effort has been made to incorporate a pairwise measure of SNPs into a global block partitioning method.

In an earlier effort to discuss how well LD patterns are consistent with block boundaries, Wall and Pritchard have evaluated three measures, namely "coverage", "absence of holes", and "non-overlapping blocks" [27]. "Coverage" refers to the portion of each chromosome which is covered by haplotype blocks. Two haplotype blocks overlap if their boundaries cross each other. A "hole" in a haplotype block occurs where a SNP is not in strong LD with any of other SNPs in the block. These features have been assessed only for the haplotype blocks produced by Gabriel's method. Obviously, all global partitioning approaches produce non-overlapping blocks which together completely cover the genome.

The block partitioning approach we present in this work consists of two steps. First, an association index is derived that characterizes each pair of SNPs as "associated", "independent", or "not-statistically-significant". Then, haplotype blocks are determined such that the number of associated SNP pairs within each block is maximized, while a limitation is set on fraction of acceptable independent SNP pairs. An iterating search algorithm is used to find the solution of the constrained optimization. The method produces global partitioning of chromosomes. Our method results in complete coverage, no overlapping, and "absence of holes" in blocks.

Having applied the method, we comprehensively compare its performance with some previously reported methods for haplotype block designation. Robustness of each method is assessed by evaluating the consistency of block boundaries on permuted samples. We also assess the potentials of block structures created by each method to serve as a reference block structure for genome-wide disease association studies. In this regard, they are assessed under conditions of use of different marker density. Finally, we evaluate whether our haplotype block partitioning method can be used to detect recombination hotspots in the genome.

## Methods

### Association test for SNP pairs

In this section, we discuss on the use of Fisher's exact test on quantifying the concept of "strongly associated" SNPs. The Fisher's exact test is a test of independence, mostly used on 2 × 2 contingency tables [28]. Despite tests based on *r*^2 ^which are approximately assessed by chi-square distribution, Fisher's exact test provides an exact probability of rejecting the null hypothesis (test of independence), observing the samples. There are many concrete applications of Fisher's exact test and its related statistics in molecular biology and genetics [29,30].

Assume that *n *samples of *L *bi-allelic sites are available. We assign a 0/1 random variable to each SNP.

Let (*X*_*i*_, *X*_*j*_) be the joint random variables denoting SNP pair (*i*, *j*). The statistic of Fisher's exact test is defined by



where *n*_*ab *_is the number of outcomes of *ab *for *X*_*i*_*X*_*j*_, *n*_1· _= *n*_10 _+ *n*_11 _and *n*_·1 _= *n*_01 _+ *n*_11_. In this context, it can be simply shown that *F*_*ex *_depends only on *n*_11_, *n*_1· _and n_·1_; i.e. *F*_*ex *_= *F*_*ex*_(*n*_11_; *n*_1·_, *n*_·1_).

We apply one-tailed *p*-value with mid-*p *correction to measure the significance of independence tests [31,32]. To do so, we separate the probability space of the null hypothesis into two subspaces of positively and negatively co-varying samples which correspond to *D *> 0 and *D *< 0, respectively. The one-tailed *p*-value subjected to this assumption can be defined by



where *n*_max_= *argmax*_*i *_*F*_*ex*_(*i*; *n*_1·_, *n*_·1_) and corresponds to the most balanced contingency table in which the sign of *D *changes.

We speed up the computation of *p*-value of association tests by establishing a table of precomputed *p*-values for every triple (*n*_1·_, *n*_·1_, *n*_11_), for each *n*_1· _= 1,..., ⌊*n*/2⌋, *n*_·1 _= 1,..., *n*_1·_, and *n*_11 _= 1,..., *n*_·1_. By table look-up and taking the symmetry into account, *p*-values of association tests of all SNP pairs in the genomic region of interest are obtained.

### Characterizing SNP pairs based on association test

Obviously, the significance increases when the size of sample is increased. In other words, when the sample size is increased then interval estimates become shorter as the *p*-value becomes smaller. Taking these into consideration, we classify all SNP pairs into three classes; "associated", "independent", and "not-statistically-significant". For given predetermined  and *p*_0 _we define those pairs with |*D'*| < as independent and other pairs as associated but if |*D'*| >  and *p-val*_one-tailed _> *p*_0_, simultaneously, we count a not-statistically-significant SNP pair.  is the least value of all |*D'*| for which SNP pairs could be assumed as associated and *p*_0 _is the level of significance of the test of independence. Choosing the proper value for  essentially depends on genetic features of population. However, choosing a stringent cut-off for *p-val*_one-tailed _makes the choice of  less strict.  = 0.8 and *p*_0 _= 0.01 are our default setting for these thresholds.

## Maximizing associated SNP pairs subject to limited independent pairs

Given haplotype samples, all pairs of SNPs are classified as associated, independent, and not-statistically-significant pairs in the whole region. It is usually convenient to avoid extra computation by setting a maximum physical distance above which no linkage is assumed between SNPs. For instance, markers 500 kb from each other are usually assumed independent by some researchers. Toward an objective definition for haplotype blocks, we assume that in a population away from genetic drift, selection force, and migration, a haplotype block eventually determines two boundaries on the genome within which every SNP pair is in "association". A similar idea has been recently considered by Pattaro et al. [33], though in their own approach, a likelihood model for the LD pattern in haplotype blocks is introduced in which two distinct distributions model independent and associated SNPs, separately.

Many SNP pairs may be identified as independent pairs in a block, basically because not all existing variations are available and the limited samples from the population haplotypes may not be adequate to estimate the real situation. Therefore, we model the problem as finding a block partitioning such that the most possible number of associated SNP pairs are included in blocks while independent SNP pairs within blocks are kept limited.

Like other multi-objective problems, there is a trade-off between achieving blocks including maximum associated SNP pairs and blocks with minimum independent SNP pairs. The former suggests haplotypes as large as whole chromosome while the latter results in single SNP blocks. To address this issue, we model the problem using a constrained optimization in which both objectives are involved. Formally, we define the problem as,



where *A*[*a*, *b*] and *B*[*a*, *b*] are the numbers of independent and associated SNP pairs in the genome segment between SNP *a *and SNP *b*, respectively. The maximization is taken over all partitioning sets such that 0 = *s*_0 _<*s*_1 _< ⋯ <*s*_*k *_= *L *are (unknown) indices of SNPs at left edges of (unknown) *k *blocks. *N*_*ind *_is the number of independent SNP pairs in entire genomic region and *α *is an arbitrary constant between zero and one that denotes the largest tolerable fraction of independent pairs in blocks.

To solve the proposed problem, we convert the constrained optimization problem to an unconstrained problem by using a Lagrange multiplier, as follows,



Where *λ *is an unknown real positive parameter associated with *α*. Given a fixed value for the Lagrange multiplier, the reduced problem can be solved by a conventional dynamic programming approach as,



where *S*(*i*; *d*) = *B*[*i *- *d *+ 1, *i*] - *λA*[*i *- *d *+ 1, *i*] is the score of the genomic interval ending at SNP *i *and consisting of *d *SNPs and *S*^*opt*^(*i*) is the score of optimum block partitioning for *i *leading SNPs. Arbitrarily, the maximum number of SNPs within a block can be set to *w*. To obtain a proper value for Lagrange multiplier, we apply a binary search procedure in which reduced problem with respect to different values for *λ *is iteratively solved until the desired constraint on the number of independent SNP pairs within blocks is satisfied. In general, increasing *λ *decreases the sum of independent SNP pairs included in blocks. In our experience and when *α *= 0.01, the Lagrange multiplier is obtained by about 10 iterations.

### An alternative algorithm based on Gabriel's index

In fact, our approach can be applied to improve any method that introduces haplotype blocks based on some pairwise index for SNPs. For instance, Gabriel's method [26] also introduces a three state index for SNP pairs based on the confidence interval of *D'*. None of the previous methods in this category incorporates any global optimization on block partitioning.

We have developed other haplotype block partitioning using the above optimization scheme substituting the Gabriel's index as SNP pair characterization. Both varieties of our method, one based on the association index derived from the Fisher's exact test and the other based on Gabriel's index have been incorporated in our extension to the open source widely accessed software, Haploview ver. 4. The software is running under JAVA and is publicly available via .

To deal with unphased genotype data, our method is assisted by the "two loci genotypes" phasing approach as implemented by [11] in Haploview. This approach is a simplified EM algorithm to infer frequencies of four possible alleles on two loci. Detailed formulas for this preprocessing can be found in [12].

### Method Comparison

We compare our proposed algorithm with other methods of haplotype block partitioning based on some descriptive aspects of haplotype blocks, performance on a block-based case-control study, and detecting recombination hotspots. In addition to the new methods introduced in the present paper, we choose six other available haplotype block partitioning algorithms. Table 1 summarizes the main features of these methods in our trial.

In Table 1, HOT is an exception. It has been developed as a method for inferring recombination "hotspots" throughout the human genome. However, a region between two consecutive hotspots can also be considered a haplotype block because the recombination level in the region is relatively low. In addition, comparison between other methods and HOT may suggest clues on extending application of block partitioning methods to recombination hotspot detection.

There is another method, MB, which has not been explicitly introduced in the literature. It is a special option in the HapBlock software in which for each block, exactly one tagSNP is assumed. Therefore, this algorithm finds the least possible number of haplotype blocks covering the genome while satisfying the haplotype diversity criterion.

### General aspects of haplotype block partitioning

Our first study concerns examining some general aspects of haplotype blocks in a real sample of haplotypes. We obtained the haplotype sample of the CEU population from HapMap database, release 22, on ten ENCODE regions. Through the HapMap Project [34], dense genotype data for ENCODE regions have been published. These ten regions have been selected by the Encyclopedia of DNA Elements Project [35] as the pilot phase to identify the functional elements of human genome. Table 2, adapted from the HapMap website, summarizes the genomic information of ten ENCODE regions and the number of assayed SNPs in CEU panel by HapMap.

**Table 2:** HapMap ENCODE samples used for haplotype block partitioning.

Region name	Chromosome band	Genomic interval (NCBI B36)	Genotyped SNPs*
ENr112	2p16.3	Chr2:51512208..52012208	2,601
ENr131	2q37.1	Chr2:234156563..234656627	2,214
ENr113	4q26	Chr4:118466103..118966103	2,538
ENm010	7p15.2	Chr7:26924045..27424045	1,830
ENm013	7q21.13	Chr7:89621624..90121624	1,770
ENm014	7q31.33	Chr7:126368183..126865324	3,343
ENr321	8q24.11	Chr8:118882220..119382220	2,128
ENr232	9q34.11	Chr9:130725122..131225122	1,909
ENr123	12q12	Chr12:38626477..39126476	2,189
ENr213	18q12.1	Chr18:23719231..24219231	1,990

Adapted from  (retrieved on 20 Jun 2008).

* Genotyped SNPs in CEU panel.

There are about 2000 SNPs assayed in each ENCODE region in the CEU panel. However, we reduced SNPs to those which are commonly ascertained for all three HapMap panels, CEU, YRI, and JPT+CHB. Moreover, for each region, we drew out the top 400 SNPs ordered by heterozygosity out of the whole region. To do so, we divided the region of interest into 20 equal-length subintervals and then for each one, we picked the 20 most heterozygous SNPs from the SNPs shared in all panels. Therefore, a nearly uniform distribution of the most "informative" SNPs was obtained. This preliminary reduction was necessary for some of the block partitioning methods, as they can not achieve the result for a huge sample size in a reasonable time. We apply all methods of Table 1 for block partitioning to these data. In our study, we first examine the resulting haplotype blocks for haplotype diversity and htSNP coverage.

To measure haplotype diversity, we apply a clustering approach that is a simple generalization of the commonly used definition of "common haplotypes" introduced by Patil et al. [1]. For each block, we group haplotypes into the same cluster such that every two members differ in at most four percent of SNPs. This fine, yet nonzero tolerance, resolves the ill effects of random noises and/or wrongly-assayed SNPs in estimation of the haplotype diversity in long length blocks. The clusters with six or more haplotypes are considered as non-occasional clusters and indicate significant polymorphisms in the population. By common haplotype coverage, we mean the fraction of whole sample which belongs to any non-occasional cluster.

The consistency of haplotype blocks with the pattern of LD would be also appealing. Intuitively, a hole in a haplotype block is where an SNP has no significant association with other SNPs of the same block [27]. In a similar way, we call cases of SNPs that are in strong association with SNPs of other blocks islands. Precisely, we count an SNP as a hole if its intra-block average |*D'*| is less than 0.8 and as an island if its inter-block average |*D'*| is greater than 0.8.

It is a common question to what extent different methods would recognize similar haplotype blocks. The usual approach considers the sum of distances between "corresponding" boundaries in two different block partitionings. While it seems an intuitive measure to find block similarities but determining which boundaries are in correspondness does not have a straightforward rule, because two different partitioning configurations usually have different number of blocks and block boundaries might be often far apart from each other. In such cases, we propose another similarity measure by the following expression,



where *L *is the number of SNPs in the whole region, *k *is the number of blocks of *P*_1 _∪ *P*_2_, and *l *is the number of SNPs in each block of the union partitioning. This measure shows the fraction of SNP pairs which are commonly included by both *P*_1 _and *P*_2_.

### Robustness of block partitioning methods

**Figure 1:**
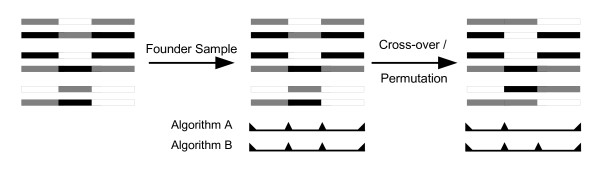
**Robustness of haplotype block partitioning**. To assess the significance of a haplotype block partitioning algorithm, assume that the given samples establish a "founder" group apart from the main population. Both algorithms A and B find the same boundaries for haplotype blocks upon the "founder" sample (middle). If the population size is kept fixed, no mutation occurs and cross-overs happen only on boundaries of the blocks then after many generations all genotypes within the initial blocks stay the same while two locus alleles of SNP pairs between different blocks change. This results in different blocks by Algorithm A and the same block partitioning by Algorithm B (right). We call a block partitioning "robust" if the method reports the same block structure for haplotypes many generations after the "founder" haplotypes.

A simplified explanation for existence of blocks is that recombination events in ancestral generations predominantly occurred at block boundaries, and not within blocks. As such, observed block boundaries may be taken as hotspots of recombination. Based on this model, a robust block partitioning algorithm will define the same block boundaries whether applied to data of an ancestral generation or to data of a recent generation. The preservation of boundaries by various block partitioning methods can be checked by comparing the boundaries produced at generation one and boundaries produced some generations later (Figure 1). For this purpose, 120 HapMap 9q34.11 haplotypes were followed by simulation through ten generations, assuming crossover probability of 0.5 at the boundaries per generation and a fixed population size. This process was repeated 500 times for each method and the configuration of blocks obtained in each iteration was recorded to assess robustness of block partitioning algorithms.

### The htSNP coverage

To obtain the tagging SNPs that are required to describe all haplotype variations within a block, we employed the htSNPer software [36]. The htSNPer software incorporates an efficient branch and bound algorithm to find the exact solution of the minimum htSNP selection problem. We used default settings of htSNPer, i.e. htSNPs were defined to cover 80% "common haplotypes" and the threshold for common haplotype frequency was set to 0.05. However, we changed parameters of its block partitioning subroutine such that it does not function. This setting allows the minimum set of haplotype-tagging SNPs to be obtained for each haplotype block of each method. Recall that resulting htSNPs are mainly affected by the shape of underlying block partitioning.

Given a set of htSNPs, we find the largest vicinity on the chromosome within which only one htSNP is enough to capture all haplotype variations. Then, we repeat the procedure on remaining regions by two, three, and more htSNPs until the entire data is covered. We refer to the length of total genomic segments covered in the *k*-th step of this procedure as "*k*-htSNPs coverage". We compare methods of haplotype block partitioning with regard to this measure, too.

### Application of haplotype blocks in disease association studies

The performance of block-like models of genomes in recognizing trait-associated loci can be assessed through a case-control design. A plan of experiment can include the following steps: 1) Using available haplotype data in HapMap, the block structure of chromosomes in a certain population is determined. 2) Considering the genotyping cost and practical limitations, an efficient number of SNPs is selected as markers to assay genotypes of case and control samples in these loci, as phase I genotyping. 3) An association test is performed on each block to obtain a scan of probably trait-associated blocks over the map. 4) More SNPs in those probable blocks of the previous step are genotyped until the desired fine map is achieved. Compared to the frequently used method of sliding window, this approach has two advantages. First, it needs a lower cost for genotyping. Secondly, there is not a common agreement on the selection of an optimum window.

To evaluate the performance of various models for block partitioning in case-control studies, we apply the first three steps of the block-based design as mentioned above on simulated data. We consider two additive single locus disease models with GRR1 = 3 and GRR1 = 5. Since we have obtained haplotype blocks on samples taken from HapMap CEU panel, it is necessary to make sure that simulated samples have the same genetic structure as the base data. The software *gs *[37] enables us to generate genotype samples for case-control studies, using real genotype data under the desired disease models. Applying the extension model of the software, we generate 500 sets of samples, each on consisting 50 case and 50 control genotypes. We repeat the simulation for low and moderate disease allele frequencies, independently. Before the next experiments, we remove the causative SNP from each sample.

Clustering haplotypes in each block, we perform both association and significance tests by applying the Pearson chi-square statistic. The clustering algorithm is the same as the one used to define "common haplotypes". In other words, after clustering each haplotype category consists of haplotypes no two of which differ from each other in more than four percent of their SNP genotypes.

We follow two policies to select those markers needed for the phase I genotyping. In the first policy, we choose the first SNP out of every *k *consecutive SNPs. Following Carlson's approach [21] for tagSNP selection, in the second policy, we prioritize SNPs in each block based on their orders in Carlson's algorithm. We select SNPs from each block based on their order until the required number of SNPs is taken from the whole region.

Our objective is to compare the power of different algorithms under the condition that false discovery rates of all algorithms are the same. We set this common rate to 10%, as we have observed that lower levels result in unacceptably weak power in all methods (not shown). We use half the 500 sets of simulated case-control samples to find the proper *p*-value threshold corresponding to the 10% false discovery rate for each method. In details, chi-square values are obtained for blocks of each method. By the result, we can estimate the distribution of the chi-square statistic for each method. To obtain the desired *p*-value threshold, we find the *p*-value corresponding to the first decile of chi-square values of blocks which do not include the trait locus. Once the *p*-value threshold is obtained for the respective algorithm, we perform the association test on the remaining 250 case-control sets and assess the statistical power. For a better comparison, we also examine the method of single site association test, besides the block-based association test.

### Performance on detecting recombination hotspots

Our third assessment is on the application of haplotype block partitioning algorithms for detecting recombination hotspots. Since there is no consensus on recombination hotspots within real haplotype data, we apply the msHOT software [38], to simulate haplotype samples. This software is an extension of Hudson's algorithm [39] and generates samples under the coalescent model with recombination. We generate 1000 sample sets, each one includes 40 haplotypes of 300 SNPs. Other samples with 100 haplotypes in each set are also generated. Conditions set include setting hotspot region lengths at a maximum of 2 kb, a maximum of six hotspots per region of 300 kb, and a recombination rate of 50 to 400 times higher than the background rate at hotspot regions of recombination. The frequency of hotspots in the simulations was based on available knowledge of such features in the human genome [4]. The positions of the hotspots observed in the different simulations are recorded. All the block partitioning algorithms being considered are then applied to these sample sets.

To assess the performance and accuracy of haplotype block partitioning methods on detecting recombination hotspots, we counted the times that haplotype block boundaries and hotspot regions coincided with each other. Block boundaries that occur outside hotspot regions are regarded as false positives, while hotspot regions not positioned at block boundaries are regarded as false negatives. In the latter case, we consider 2 kb flanking intervals around block boundaries as a standard extent of the hotspot region. We refer to the sum of the false positive and the false negative rates as total error rate in hotspot detection. We define the ratio of hotspot regions coinciding with block boundaries to the number of all hotspot regions as the power.

## Results and discussion

### Blocks of HapMap ENCODE regions

Results obtained by eight different haplotype block partitioning methods on HapMap data in ten ENCODE regions have been summarized on Table 3. The common haplotype coverage as described in Method Comparison, denotes the fraction of haplotype sample that is covered by non-occasional haplotype clusters. Recall that we allow a small tolerance such that the haplotypes having few different alleles have been clustered into the same group. Therefore, the expected common haplotype coverage for methods like MB and HB are higher than the default 80% threshold that such methods are assuming for the haplotype block definition (Table 3). Even for other methods that are not subjected to such a diversity criterion, the level of common haplotype coverage is satisfactory. In particular, MDL, GAM and GAB usually produce short blocks that result in covering almost the whole region by common haplotypes. In contrast, our new methods, GPF and GPG, produce much wider blocks which still show reasonable common haplotype coverage.

**Table 3:** Features of different haplotype block partitioning methods on haplotype samples in ENCODE regions.

	HOT	MB	HB	MDL	GAM	GAB	GPG	GPF
Average block length (kbp)	68.9	46.8	36.2	17.1	13.3	23.3	35.7	39.7
Average block length (SNP)	52	36	27	13	10	18	27	30
Total run time (sec.)	N/A	22	743	3295	112	143	5	5
Common haplotype coverage	0.67	0.89	0.91	0.96	0.96	0.93	0.88	0.87
Hole freq.	0.50	0.23	0.14	0.06	0.04	0.04	0.15	0.18
Island freq.	0.08	0.10	0.09	0.17	0.19	0.11	0.06	0.07
Robustness	N/A	92.0	69.2	99.4	99.7	100	100	97.6

Result summary of haplotype blocks obtained by different methods on HapMap haplotypes of CEU in ENCODE regions. Common haplotype coverage denotes the fraction of all chromosomes that are covered by common haplotype variations in blocks. Hole freq. and Island freq. show the probability of a hole in blocks and the probability of an island out of any block. Robustness shows the probability that any block boundary is placed at the same location during resampling. See Table 1 for method abbreviations.

The consistency between block partitioning and LD pattern on ENCODE regions can be shown by frequencies of "hole" and "island". In general, it is expected that the partitioning with wider blocks may include more holes in blocks and smaller blocks may miss more islands. Considering these measures, methods GPG, GPF, and HB seem to be more reliable when most information of the LD pattern is to be maintained. In contrast, GAB, GAM, and MDL produce firmer blocks.

**Figure 2:**
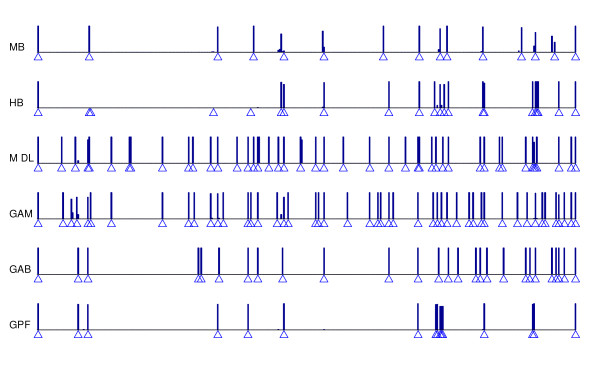
**Haplotype blocks in 9q34.11 (ENr232)**. Haplotype block boundaries obtained by several block partitioning methods on region 9q34.11. See Table 1 for method abbreviations. Locations of block boundaries in the initial sample are shown by small triangles. For each method, the order of haplotypes in each block are shuffled using a simulation of fixed population size with recombinations only in block boundaries. Generated samples are then applied to this method. The height of vertical bars depict the times that a location occurs on block boundaries.

Figure 2 illustrates the distribution of block boundaries along the chromosomal region 9q34.11 (ENr232). It shows that the number of blocks and their positions are not quite the same among the different methods used, but there are some locations that are shared by all methods. The block similarity of eight block partitioning methods are shown in Table 4, in details. Most similar methods are variants of the same method; GPG and GPF are of global partitioning based pairwise LD of SNPs, and MB and HB optimize arbitrary goals subject to haplotype diversity. Results also show that overall, GAB has the most similarity with any other method.

**Table 4:** Block similarity among several methods of haplotype block partitioning.

	HOT	MB	HB	MDL	GAM	GAB	GPG	GPF
HOT	1.00	0.32	0.31	0.16	0.13	0.26	0.39	**0.40**
MB	0.32	1.00	**0.65**	0.36	0.30	0.58	0.60	0.56
HB	0.31	**0.65**	1.00	0.41	0.35	**0.60**	0.56	0.54
MDL	0.16	0.36	0.41	1.00	**0.54**	0.42	0.30	0.29
GAM	0.13	0.30	0.35	**0.54**	1.00	0.41	0.25	0.24
GAB	0.26	0.58	**0.60**	0.42	0.41	1.00	0.57	0.55
GPG	0.39	0.60	0.56	0.30	0.25	0.57	1.00	**0.88**
GPF	**0.40**	0.56	0.54	0.29	0.24	0.55	**0.88**	1.00

The similarity measure indicates the proportion of SNP pairs commonly shared by different partitionings on ten ENCODE regions. See Table 1 for method abbreviations.

**Figure 3:**
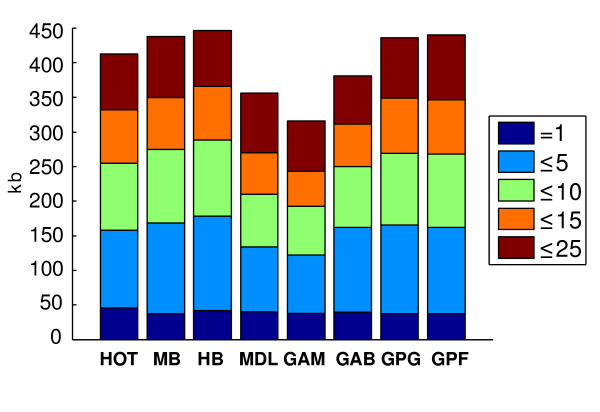
**Distribution of genome length covered by htSNPs**. The height of each stacked bar at each level shows the total length of regions in the genome which are tagged by a certain number of htSNPs. See Table 1 for method abbreviations.

The last row of Table 3 shows how much block partitioning methods produce consistant block boundaries when the resampling is performed. All methods except for HB are quite robust under permutation. The poor result of HB can be explained as an artifact of its underlying optimization approach. Usually, the high sensitivity is a consequence of the optimality. As shown in Figure 2, some blocks obtained by HB have been merged into one block on resampled generations. It shows that the model of minimal set of tagSNPs may ignore the essential structure of haplotypes data. In contrast, robustness of Gabriel's method is surprising. Both methods GAB and GPG using the Gabriel's association index are perfectly robust. Using the real haplotype samples, the htSNP base coverage has also been computed for each method. Figure 3 illustrates the average htSNP base coverage averaged over ten ENCODE regions. In addition, for each ENCODE region separately, the number of htSNPs is shown in Table 5. As described in the previous section, with inclusion of blocks that need progressively larger number of htSNPs in order to be identified, a complete coverage of the chromosome can potentially be achieved. It is observed that in all the methods the major coverage encompasses regions defined by 2–5 htSNPs. It should be noted that the method used for block partitioning affects the haplotype diversity and consequently the number of tagging SNPs. Generally, larger htSNP coverage produces a more economical genotyping. Here, we observe that HB has the best htSNP base coverage in every level. This is not unexpected because its algorithm has been specially tailored for this purpose. As shown by the last segments of bars in Figure 3, methods producing smaller blocks do not reach the coverage achieved by other methods. By contrast, the difference at the start level – the genome coverage by a single htSNP – seems not to be considerable among different methods. By increasing the number of htSNPs, a greater difference in the covered lengths can be observed. GAM and MDL demand more htSNPs for more complete coverage. GAB results in the best coverage among the three local partitioning methods, even by few numbers of htSNPs. It is an undeniable advantage for MB which produces the htSNP coverage very similar to the best method, HB, while its optimized objective is much less complicated than HB and attained by few computations.

**Table 5:** Number of htSNPs in each ENCODE region.

region name	chromosome band	HOT	MB	HB	MDL	GAM	GAB	GPG	GPF
ENr112	2p16.3	37	35	33	51	96	77	32	33
ENr131	2q37.1	32	48	40	60	101	79	47	42
ENr113	4q26	34	37	30	46	67	47	31	31
ENm010	7p15.2	36	37	34	49	87	78	37	36
Enm013	7q21.13	17	16	15	34	69	29	23	25
Enm014	7q31.33	38	27	25	48	67	47	27	27
ENr321	8q24.11	27	35	26	49	63	49	31	30
ENr232	9q34.11	51	47	42	63	70	75	49	52
ENr123	12q12	14	33	29	48	79	59	37	38
ENr213	18q12.1	31	36	30	52	69	46	28	33

For each block, the minimum number of htSNPs has been obtained by htSNPer. The number of htSNPs in the whole region is the sum of the number of htSNPs in all blocks. See Table 1 for method abbreviations.

**Figure 4:**
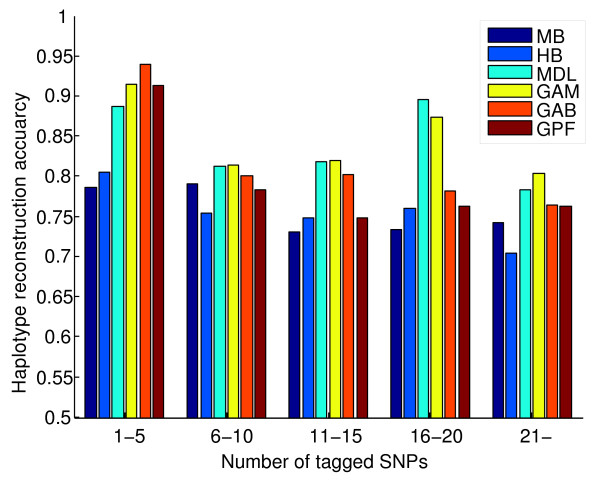
**Accuracy of haplotype reconstruction by htSNPs**. The accuracy of haplotype reconstruction shows the probability that a haplotype out of a given sample can be perfectly identified when information of htSNPs is exclusively available.

By comparing the result of htSNP selection for each ENCODE region (Table 5), we found that relaxing the constraint of haplotype diversity for block definition can potentially result in fewer htSNPs. This proposition can be verified by comparing the result of GPF and GPG with HB in Table 5. However, it has been reported that carrying much genomic information by the least possible number of tagSNPs, while appealing in reducing genotyping cost, can result in less accurate repeatable findings [40]. To address this issue, we examine the efficiency of the selected htSNPs in haplotype reconstruction, to find out whether the number of htSNPs in large blocks is underestimated. We performed a perfect cross-validation procedure for each method to assess accuracy of haplotype reconstruction based on HapMap haplotype data in all ENCODE regions. As shown by Figure 4, htSNPs in every method can describe all the necessary information to reconstruct at least 70% of crossed out haplotypes from given samples. It is close to the number that htSNPer guarantees to cover for "common haplotypes". The reconstruction accuracy varies among different block partitioning methods, but in general, it slightly decays when tagged SNPs increase. The small blocks of MDL and GAM have a greater effect on the accuracy as it seems hard to find a trend for accuracy decay in Figure 4 for these methods. Reconstruction accuracies of other methods are almost steady after six or more SNPs being tagged by a htSNP.

### Effect of different block structures on performance of disease association study

Values of Type I error on Table 6 show the probability that the test recognizes a block as trait-associated when the block does not actually include the trait locus. These errors are results of chi-square tests at a 0.05 level of significance, which have been performed on 250 sets of simulated case-control samples before taking adaptive thresholds for each method (see Method section). As shown in Table 6, with higher risk ratio, more type I error is committed by all methods. However, disease allele frequency has a greater effect on type I error than GRR1.

**Table 6:** Type I error in the disease association study.

Disease model parameters	SS*	MB	HB	MDL	GAB	GPG	GPF
DAF = 5% – 15%, GRR1 = 3	0.26	0.20	0.17	0.16	0.17	0.13	0.13
DAF = 5% – 15%, GRR1 = 5	0.32	0.24	0.21	0.20	0.22	0.16	0.17
DAF = 20% – 30%, GRR1 = 3	0.29	0.21	0.18	0.17	0.19	0.14	0.15
DAF = 20% – 30%, GRR1 = 5	0.34	0.25	0.22	0.22	0.23	0.17	0.19

Chi-square tests have been performed at 0.05 level of significance, on 250 sets of 50/50 case-control samples. Each entry depicts the proportion of blocks that have been falsely recognized as trait-associated to all the blocks that do not include the trait locus. * Method of single site test. See Table 1 for method abbreviations.

**Figure 5:**
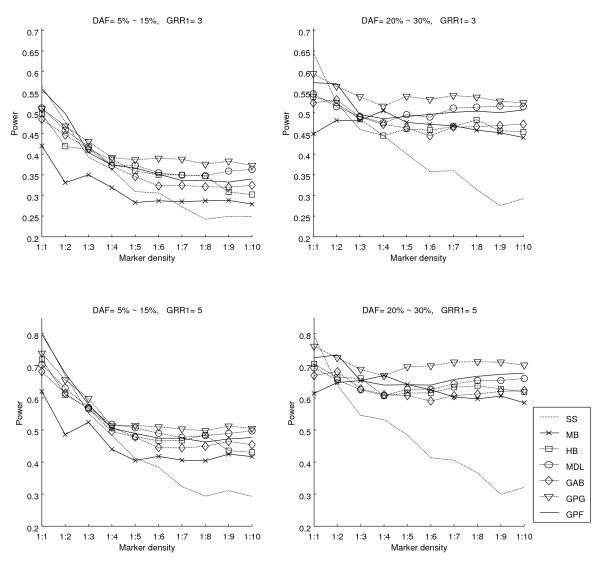
**Performance of various block structures on disease association study with non-prioritized SNPs**. Power of a block-based disease association test vs. density of marker distribution for various block structures is shown. SS is the method of single site test. For other abbreviations see Table 1. No particular policy has been applied to select SNPs for marker locations.

**Figure 6:**
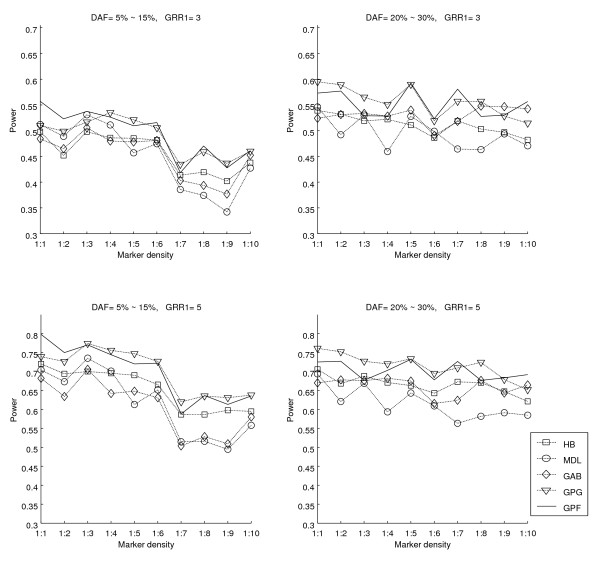
**Performance of various block structures on disease association study with prioritized SNPs**. Power of a block-based disease association test vs. density of marker distribution for various block structures is shown. SS is the method of single site test. For other abbreviations see Table 1. Sorting SNPs based on their informativeness in each block, a number of SNPs proportional to the marker density and size of the block is selected.

The power of the block-based association test and the single site method are depicted in Figures 5 and 6. The power of single site test decreases with sparser marker distribution. The same behavior can be partially found in block-based methods. However, the power of block-based methods is generally higher than the single site method even in the case of lower marker density. It shows that incorporating the LD information into the association study results in better performance. In fact, in the case of small disease allele frequency, the decrease of power is due to weaker LD between causative SNP and other SNPs. As shown in Figure 5, our methods are slightly more successful than other methods in improving the power of association tests.

Selecting marker positions by SNPs that have been ordered based on "informativeness" results in lesser decrease in the power of methods where marker distribution has lower density. In the case where SNPs are selected by prioritizing, the power of methods remains high, even when only one fifth of the original SNPs are used as markers (Figure 6). MDL is relatively efficient when markers are selected uniformly. When markers are prioritized, as one might expect, HB performs better. Nevertheless, the two versions of our model – GPG and GPF – are more efficient even when marker density is low.

### Performance on recombination hotspot detection

Given arbitrary hotspot regions, the sample generator program provides us with two datasets of simulated haplotype samples. Using the hotspot locations as our references, we can estimate the hotspot detection error. This error is the sum of false positive and negative hits. Table 7 illustrates the total error rate in recombination hotspots detection with respect to different sample sizes (40 and 100), for each block partitioning method. Except for HB, the error rate of other methods is reasonable. The method structure in MB and HB is the same, but the former minimizes the number of haplotype blocks and the latter minimizes the number of haplotype-tagging SNPs. Therefore, it can be deduced that the approach of minimal tagging does not fit well with patterns of recombination. At the opposite side, GPG and GPF get the least total error rate among other methods.

**Table 7:** Total error rate on detection of recombination hotspots*.

		MB	HB	MDL	GAM	GAB	GPG	GPF
N = 40	false positive rate	2.6	15.4	3.8	5.3	3.0	1.9	1.6
	false negative rate	2.5	0.9	1.2	0.7	3.1	0.9	1.0
	total error rate	5.1	16.3	5.0	6.0	6.1	2.8	2.6

N = 100	false positive rate	3.8	12.2	2.6	4.9	3.4	1.1	1.0
	false negative rate	2.2	1.1	0.9	0.9	2.2	0.8	1.1
	total error rate	6.0	13.3	3.5	5.8	5.6	1.9	2.0

See Table 1 for method abbreviations. N is the number of haplotypes. * All values are in percent.

**Figure 7:**
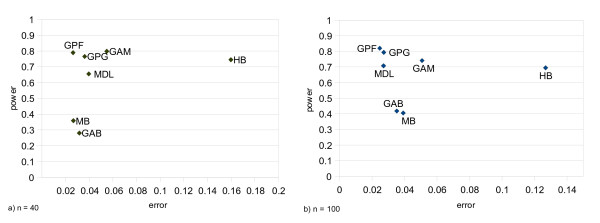
**Performance on recombination hotspot detection**. Power indicates the rate of coincidence between hotspots and block boundaries. "Error" is the probability that a location out of any hotspot regions is identified as a block boundary by the method. The assessment has been performed on two sets of simulation generated samples with 40 haplotypes in each replication, (a), and with 80 haplotypes in each replication, (b).

It seems more probable that a block boundary occurs out of hotspot regions than a hotspot region is left undetected with no block boundary. Figure 7 plots the power of the hotspot detection versus the error, i.e the false positive rate. In general, increasing haplotype samples reduces the error rate by all methods but MB (See Table 7). However, improvement in the performance of MB compensates for this effect. For GPG, MDL, GAB and MB the power increases by increasing the sample size. By contrast, for GPF, HB and GAM, we observe a decrease in power, but this is accompanied by a better prediction accuracy. Our proposed method has the lowest type I error among the other methods and still, it correctly detects a hotspot with ~75% probability. GAM also shows the same performance, around 80 percent. This is mainly due to its block definition that is relevant to the coalescent model of the simulated data.

## Conclusion

Here, we present a method for global haplotype partitioning based on pairwise analysis of SNPs. In this approach, haplotype blocks are defined such that the number of associated pairs in blocks is maximal, and blocks include only a small number of independent SNP pairs. The normalized coefficient of linkage disequilibrium, *D'*, is used as a scan statistic to determine independent SNP pairs and Fisher's exact test and its corresponding *p*-value determine the significance of dependency between SNP pairs. Furthermore, Gabriel's index is applied in determination of association classes.

Since the early observation of haplotype block structure in human genomes, several groups have developed block models assuming constraints on haplotype diversity. However, it has been suggested that such assumptions should be used carefully in applications [41,42]. Our results from ENCODE data show that methods based on pairwise analysis of SNPs, without initial assumptions on haplotype diversity, find blocks in which haplotype diversity is consistent with the standard thresholds used in classical methods. We assessed the similarity of haplotype block structures by counting SNP pairs in overlapping regions in blocks of different partitionings. We did not find any general concordance among block boundaries in different methods. A previous study has also reached the same conclusion [24]. Nevertheless, each method does produce blocks with 50% similarity with blocks of at least one other method.

The consistency of block boundaries within each single method was also investigated by a permutation resampling. To do so, we recorded the number of times in which a certain method would reproduce the same boundaries when applied to simulated recombinant samples. It was observed that the rule of Gabriel to determine the association index within SNP pairs was highly robust. Our algorithm was also relatively robust.

In our method, the number of htSNPs is not subjected to minimization. However, the number and also the coverage of htSNPs within the resulting blocks compare well with the optimal values obtained by diversity-based approaches.

In a case-control study, a block-based approach for mapping a single locus trait was applied to blocks of various methods. The results show that any block-based association test is considerably more efficient than the conventional single site association test. In particular, our newly developed block partitioning method performed best accuracy for the case-control study, even when a low marker density is available.

Biological considerations suggest that block boundaries produced by block partitioning methods should exhibit some concordance with recombination hotspots. In this regard, we assessed the performance of methods on simulated data. Global block partitioning methods performed best both in terms of accuracy and power. In fact, our method may be considered an efficient and simple tool for gaining insight of recombination hotspots.

In conclusion, our assessments show that our proposed global partitioning method, the method of minimum description length, and Gabriel's method are all promising for case-control association studies and for detection of recombination hotspots. Furthermore, we have shown that allelic association of SNP pairs can partially describe aspects of genomic variations in human populations.

## Authors' contributions

The problem and the use of pairwise measures to depict block-like structure of sample haplotypes have been proposed by MS. The mathematical model, computer implementation and method comparison have been developed by AK. HP revised the proposed model and redefined it in statistical terms. EE rewrote the article from a draft. All authors read and approved the final manuscript.
